# Real-world Experience of Posaconazole Therapeutic Drug Monitoring in Oncology Patients: Clinical Implications of Hypoalbuminemia as a Predictor of Subtherapeutic Posaconazole Levels

**DOI:** 10.1093/ofid/ofae185

**Published:** 2024-03-29

**Authors:** Guy Handley, John Greene, Anthony P Cannella, Ana Paula Velez, Shivan Shah, Yanina Pasikhova

**Affiliations:** Division of Infectious Disease and International Medicine, Department of Internal Medicine, Morsani College of Medicine, University of South Florida, Tampa, Florida, USA; H. Lee Moffitt Cancer Center and Research Institute, Tampa, Florida, USA; H. Lee Moffitt Cancer Center and Research Institute, Tampa, Florida, USA; Division of Infectious Disease and International Medicine, Department of Internal Medicine, Morsani College of Medicine, University of South Florida, Tampa, Florida, USA; H. Lee Moffitt Cancer Center and Research Institute, Tampa, Florida, USA; Division of Infectious Disease and International Medicine, Department of Internal Medicine, Morsani College of Medicine, University of South Florida, Tampa, Florida, USA; H. Lee Moffitt Cancer Center and Research Institute, Tampa, Florida, USA; Division of Infectious Disease and International Medicine, Department of Internal Medicine, Morsani College of Medicine, University of South Florida, Tampa, Florida, USA; H. Lee Moffitt Cancer Center and Research Institute, Tampa, Florida, USA; Department of Pharmacy, H. Lee Moffitt Cancer Center and Research Institute, Tampa, Florida, USA

**Keywords:** hypoalbuminemia, posaconazole, therapeutic drug monitoring, invasive fungal infection, infections in oncology patients

## Abstract

**Background:**

Posaconazole maintains broad antifungal activity and is employed for prevention and treatment of invasive fungal infections in oncology patients. Older formulations required therapeutic drug monitoring, and specific plasma drug levels have been recommended. This study evaluated factors associated with subtherapeutic concentrations with the newer delayed-release tablet formulation.

**Methods:**

In this retrospective, single-center cohort study at a national comprehensive cancer center, all oncology patients receiving delayed-release posaconazole at standard dosing of 300 mg orally per day from 06/2021 to 07/2023 with plasma drug concentration evaluation were identified. Demographic, clinical, and laboratory data were evaluated to identify risk factors associated with subtherapeutic drug levels at targets of ≥1.25 µg/mL and ≥1.8 µg/mL.

**Results:**

Of 110 patients identified, 98 met criteria for inclusion in the study. The median time from initiation of posaconazole to drug level assessment was 13 days, and the median concentration was 1.29 µg/mL. Of the 22 patients receiving posaconazole for prophylaxis, 5 (22.7%) failed to achieve concentrations ≥0.7 µg/mL, and of 76 patients receiving posaconazole for treatment, 38 (50%) failed to achieve concentrations of ≥1.25 µg/mL. In multivariable analysis, albumin of ≤3 g/dL and ideal body weight ≥60 kg were found to be associated with subtherapeutic levels. For a higher target of ≥1.8 µg/mL, only albumin ≤3 g/dL was associated with subtherapeutic levels for the variables evaluated.

**Conclusions:**

A higher initial dosing strategy and therapeutic drug monitoring for oncology patients with albumin ≤3 g/dL receiving posaconazole, particularly for the treatment of invasive fungal infection, could be considered.

## Teaser

The potential impact the COVID-19 pandemic had on the spread of Candida auris is seen in the significant increase in incidence at our institution from 2019 - 2022. Targeted admission surveillance serves as a valuable tool to combat the spread of Candida auris.

Posaconazole (POSA) is a triazole antifungal medication with broad in vitro antifungal activity. Guidelines recommend its use for prophylaxis in patients with intermediate- to high-risk hematologic malignancies such as acute myeloid leukemia (AML) or myelodysplastic syndrome (MDS) and those with hematopoietic stem cell transplantation (HCT) complicated by significant graft-vs-host disease (GVHD) [1]. Originally only available as an oral suspension, it was limited by major drawbacks. These included limited bioavailability, particularly in patients with mucositis, gastrointestinal GVHD, or diarrhea and nonlinear pharmacokinetics, thus resulting in inter- and intrapatient bioavailability discrepancies [2]. Subtherapeutic POSA plasma concentrations may be associated with increased breakthrough invasive fungal infection (IFI) and contrasted with high levels in pseudohyperaldosteronism; therefore, therapeutic drug monitoring (TDM) of POSA has been recommended for POSA oral solution [3, 4]. In order to overcome these limitations, a POSA delayed-release tablet (DR-POSA) was developed that appears to be only moderately affected by food or by drugs that influence gastric acidity or gut motility; it has demonstrated linear pharmacokinetics for the tested dosage range of up to 400 mg [4].

Based on the original studies and subsequent publications, 81%–90% and 75% of patients achieve therapeutic POSA levels for prophylaxis, ≥0.7 µg/mL, and treatment, ≥1.25 µg/mL, respectively [5–7]. However, based on the possibility of target attainment for more resistant pathogens, higher drug levels have been proposed of ≥0.7–0.9 µg/mL for prophylaxis and ≥1.25 µg/mL or ≥1.8 µg/mL for treatment [4, 8]. Subtherapeutic concentrations have been associated with a higher rate of breakthrough IFIs [9]. While mortality for IFI in hematologic malignancy is high, delayed appropriate therapy has been associated with substantial further increased risks of mortality in both zygomycosis and aspergillosis [10]. For DR-POSA, time to reach steady state is ∼6 days [4]. With DR-POSA concentration does not substantially differ when evaluated before dose, at 4 and 8 hours after dosing [11]. Unfortunately, POSA levels are not readily available in many institutions and require referral to a reference lab, with an approximate turnaround time of 5 days at our institution, making real-time TDM difficult and potentially detrimental to patient outcomes.

In order to identify factors associated with subtherapeutic POSA levels in our oncology patients, we conducted this single-center retrospective study. Our overarching aim was to identify patients who could potentially benefit from higher initial dosing. Particularly, patients being treated for IFIs, who carry a high mortality in this population, were the focus of this investigation.

## METHODS

We conducted an observational, single-center, retrospective quality improvement study of oncologic patients at Moffitt Cancer Center, a National Cancer Institute–designated Comprehensive Cancer Center. All patients with a measured POSA concentration from 06/2021 to 07/2023 were identified. Adult patients receiving oral DR-POSA 300-mg/d dosing for either prophylaxis or treatment of IFI were included. Exclusion criteria included patients receiving alternative dosing or formulations, evaluation of POSA concentration before at least 5 days of therapy, evaluation within 4 hours after the most recent dose, and not actively taking POSA at the time a level was drawn. For the primary data analysis, only the patients’ initial POSA levels were included, but levels after subsequent dose adjustments were evaluated for secondary analysis to evaluate response to higher dosing. Demographic, laboratory, microbiologic, and clinical data were extracted from the electronic medical record. All patients had ≥90 days of follow-up records available.

The primary outcome was to identify risk factors associated with subtherapeutic POSA levels at targets of ≥1.25 µg/mL and ≥1.8 µg/mL in oncology patients and identify potential clinical parameters to guide consideration of potential higher upfront dosing in high-risk patients. Secondary outcomes included evaluation of patient outcomes, adverse events, epidemiology of IFI in oncology patients and subsequent POSA concentrations at higher doses, as well as evaluation of risk factors associated with subtherapeutic POSA levels at targets of ≥0.7 µg/mL. Concomitant use of interacting medications evaluated included proton pump inhibitors, histamine H2-receptor antagonists, phenytoin, rifamycins, or macrolides.

For statistical analysis, only the initial POSA concentration for each patient was included in the primary analysis. Results were presented with median and interquartile range (IQR) for continuous variables and percentages for ordinal variables. Continuous variables were compared using the Mann-Whitney *U* test, and bivariate associations were evaluated by the Pearson χ-square test, or by the Fisher exact test if there were not enough instances to perform Pearson's χ-square. A *P* value of <.05 was considered statistically significant. Variables with a *P* value <.2 were included in multivariable analysis with binary logistic regression to determine significance as well as odds ratios with 95% confidence intervals. Due to collinearity with ideal body weight (IBW), height was not included in the multivariable model. Cutoffs for albumin at ≤3 g/dL and IBW ≥60 kg were determined by classification and regression trees. Statistical analyses were conducted using SPSS Statistics, version 29.0.1.0 (SPSS, Inc., Chicago, IL, USA). This study was reviewed and approved by the University of South Florida Institutional Review Board and the Moffitt Cancer Center Scientific Review Committee.

## RESULTS

In total, 110 patients were identified, with 98 patients meeting inclusion criteria included in the study and analysis. Twelve patients were excluded based on exclusion criteria (Supplementary Figure 1). Baseline characteristics, malignancy status, indications for POSA, and infection status are included in Table 1. The median time from initiation of POSA to level assessment (IQR) was 13 (7.00–38.25) days. The median concentration (IQR) was 1.29 (0.75–1.98) µg/mL. Most (n = 76, 77.6%) patients received POSA for treatment of IFI, and of those, 29 (38.2%) met criteria for probable or proven infection based on consensus international definitions [12]. In patients receiving POSA for treatment, the median level (IQR) was 1.25 (0.74–1.89) µg/mL, with a median time to assessment from initiation (IQR) of 13 (7.00–37.00) days. Of 22 patients receiving POSA for prophylaxis, 17 (77.3%) met concentrations of ≥0.7 µg/mL. Overall, 46 (46.9%) patients failed to achieve concentrations of ≥1.25 μg/mL, and of the 76 receiving POSA for treatment of IFI, 38 (50%) failed to achieve this concentration. For the overall cohort, 67 (68.4%) failed to achieve a concentration of ≥1.8 µg/mL, and 55 (72.4%) of those receiving therapy for treatment of IFI failed to achieve this concentration. The most common site of IFI was pulmonary (63.3%). Overall, 51 (52%) patients on POSA had received allogeneic HCT, and the most common malignancy was AML (46.9%). In patients who had received HCT, 12 (23.5%) had diarrhea at the time of assessment, but of those only 2 had been diagnosed with acute gastrointestinal GVHD.

**Table 1.** Patient Baseline Characteristics, Indications, and Infection Sites

Characteristic	Total n = 98
Age, median (range), y	62.5 (20–85)
Sex, No. (%)	…
Male	33 (33.7)
Female	65 (66.3)
Race, No. (%)	…
White	84 (85.7)
Black or African American	8 (8.2)
Other or not reported	6 (6.1)
Body mass index, median (range), kg/m^2^	26.33 (16.37–41.78)
Weight >90 kg, No. (%)	28 (28.6)
Albumin, median (range), g/dL	3.1 (1.7–4.8)
Albumin ≤3 g/dL, No. (%)	47 (48.0)
Albumin >3 g/dL, No. (%)	51 (52.0)
Malignancy, No. (%)	…
Acute myeloid leukemia	47 (48.0)
Myelodysplastic syndrome	15 (15.3)
Lymphoma	9 (9.2)
Myeloproliferative neoplasms	6 (6.1)
Acute lymphocytic leukemia	4 (4.1)
Multiple myeloma	1 (1.0)
Other	17 (17.3)
Stem cell transplant and cellular therapies	…
Allogeneic	49 (50.0)
Chimeric antigen receptor–T therapy	10 (10.2)
Stem cell+ CAR-T therapy	2 (2.0)
Acute gastrointestinal graft-vs-host disease, No. (%)	10 (10.2)
Interacting drug—any, No. (%)	82 (83.7)
Proton pump inhibitor, No. (%)	51 (52.0)
Diarrhea, No. (%)	21 (21.4)
Indication for posaconazole, No. (%)	…
Treatment	76 (77.6)
Prophylaxis	22 (22.4)
Infections definition, No. (%)	…
Possible	44 (44.9)
Probable	24 (24.5)
Proven	5 (5.1)
Not applicable (prophylaxis or undefined)	25 (25.5)
Infection site, No. (%)	…
Pulmonary	62 (63.3)
Rhino-cerebral	7 (7.1)
Cutaneous	5 (5.1)
Other	2 (2.0)
Not applicable (prophylaxis)	22 (22.4)
Organism isolated, No. (%)	28 (28.6)

Factors associated with subtherapeutic levels for targets of ≥1.25 µg/mL and ≥1.8 µg/mL are presented in Tables 2 and 3, respectively. For the primary outcome of achieving a target concentration of ≥1.25 µg/mL, weight, height, IBW ≥60 kg, and albumin ≤3 g/dL were all found to be associated with subtherapeutic levels in univariable analysis. Male gender trended toward subtherapeutic levels, but this was not found to be statistically significant (*P* = .054). For a target concentration of ≥1.8 µg/mL, height, IBW ≥60 kg, and albumin ≤3 g/dL remained associated with subtherapeutic levels. Male gender was associated with subtherapeutic levels at this target (*P* = .011). In a multivariable analysis for a target of ≥1.25 µg/mL, IBW ≥60 kg and albumin ≤3 g/dL remained significant factors associated with subtherapeutic levels, and for a target of ≥1.8 µg/mL, only albumin ≤3 g/dL retained significance. Of 34 patients with IBW ≥60 kg and albumin ≤3 g/dL, only 11 (32.3%) and 5 (14.7%) patients were therapeutic at targets of ≥1.25 µg/mL and ≥1.8 µg/mL, respectively. Concomitant use of interacting drugs including proton pump inhibitors, diarrhea, acute GI GVHD, type of malignancy, and receipt of a bone marrow transplant were not found to be significant in either univariable or multivariable analysis.

**Table 2.** Risk Factors for Subtherapeutic Posaconazole Concentrations for a Target Concentration ≥1.25 µg/mL

	Posaconazole Level <1.25 µg/mL (n = 46)		Posaconazole Level ≥1.25 µg/mL (n = 52)			Multivariable Logistic Regression	
Risk Factor					*P* Value	OR (95% CI)	*P* Value
Age, median (IQR), y	63.50 (53.75–70.00)	…	62.00 (52.25–67.75)	…	0.598	…	…
Weight, median (IQR), kg	85.55 (72.07–93.75)	…	75.65 (62.72–91.55)	…	.030	0.98 (0.95–1.00)	.094
Weight >90 kg	14	30.4%	14	26.9%	.701	…	…
Height, median (IQR), cm	177.80 (170.00–182.00)	…	169.45 (160.25–177.72)	…	.003	…	…
IBW, median (IQR), kg	72.86 (65.60–76.80)	…	64.13 (54.45–72.62)	…	.003	…	…
IBW ≥60 kg	39	84.8%	31	59.6%	.006	0.19 (0.04–0.95)	.042
BMI, median (IQR), kg/m^2^	26.04 (23.78–31.34)	…	26.44 (23.31–30.19)	…	.773	…	…
Female gender	11	23.9%	22	42.3%	.054	0.54 (0.12–2.42)	.421
AML	22	47.8%	25	48.1%	.980	…	…
Myeloid malignancy	34	73.9%	34	65.4%	.361	…	…
AlloSCT	24	52.2%	25	48.1%	.686	…	…
GI aGVHD	3	6.5%	7	13.5%	.327	…	…
Interacting drug—any	39	84.8%	43	82.7%	.780	…	…
PPI	26	56.5%	25	48.1%	.404	…	…
Antacid, single agent	38	82.6%	42	80.8%	.814	…	…
Diarrhea	12	26.1%	9	17.3%	.290	…	…
Albumin ≤3 g/dL	28	60.9%	19	36.5%	.016	0.32 (0.13–0.77)	.012

Abbreviations: AlloSCT, allogeneic stem cell transplant; AML, acute myeloid leukemia; BMI, body mass index; GI aGVHD, acute gastrointestinal graft-vs-host disease; IBW, ideal body weight; IQR, interquartile range; PPI, proton pump inhibitor.

**Table 3.** Risk Factors for Subtherapeutic Posaconazole Concentrations for a Target Concentration ≥ 1.8 µg/mL

	Posaconazole Level <1.8 µg/mL (n = 67)		Posaconazole Level ≥1.8 µg/mL (n = 31)			Multivariable Logistic Regression	
Risk Factor					*P* Value	OR (95% CI)	*P* Value
Age, median (IQR), y	60.00 (53.00–68.00)	*…*	64.00 (57.00–69.00)	…	.206	…	…
Weight, median (IQR), kg	83.00 (68.20–93.00)	…	75.90 (61.10–91.70)	…	.148	0.99 (0.96–1.02)	.487
Weight >90 kg	19	28.4%	9	29.0%	.945	…	…
Height, median (IQR), cm	177.00 (168.00–182.00)	…	167.00 (160.00–177.00)	…	.004	…	…
IBW, median (IQR), kg	70.46 (62.34–76.80)	…	63.22 (52.38–72.28)	…	.003	…	…
IBW ≥60 kg	54	80.6%	16	51.6%	.003	0.26 (0.06–1.11)	.070
BMI, median (IQR), kg/m^2^	26.04 (23.49–30.30)	…	28.84 (23.71–30.55)	…	.529	…	…
Female gender	17	25.4%	16	51.6%	.011	0.90 (0.20–4.03)	0.895
AML	32	47.8%	15	48.4%	.954	…	…
Myeloid malignancy	47	70.1%	21	67.7%	.810	…	…
AlloSCT	32	47.8%	17	54.8%	.515	…	…
GI aGVHD	4	6.0%	6	19.4%	.068	3.80 (0.82–17.58)	.087
Interacting drug—any	55	82.1%	27	87.1%	.533	…	…
PPI	34	50.7%	17	54.8%	.706	…	…
Antacid, single agent	54	80.6%	26	83.9%	.697	…	…
Diarrhea	16	23.9%	5	16.1%	.384	…	…
Albumin ≤3 g/dL	37	55.2%	10	32.3%	.034	0.35 (0.13–0.94)	.036

Abbreviations: AlloSCT, allogeneic stem cell transplant; AML, acute myeloid leukemia; BMI, body mass index; GI aGVHD, acute gastrointestinal graft-vs-host disease; IBW, ideal body weight; IQR, interquartile range; PPI, proton pump inhibitor.

For the target threshold of ≥0.7 µg/mL, weight >90 kg, receipt of an allogeneic stem cell transplant, and diarrhea were the only factors associated with subtherapeutic levels in univariable analysis; however, none retained significance in multivariable analysis (Supplementary Table 1). Of 79 with levels ≥0.7 µg/mL, 38 had albumin levels ≤3 g/dL (48.1%), as compared with 9 of 19 patients with lower levels (47.4%; *P* = .954).

## DISCUSSION

This is the first study to find a specific albumin level highly associated with subtherapeutic levels for the DR-POSA formulation at certain target POSA concentrations of ≥1.25 µg/mL and ≥1.8 µg/mL, which are often used when treating IFI in oncology patients and may provide opportunities for clinical intervention. While both of these levels have been proposed as targets when treating pathogens, there is not a consensus, and larger studies are required for validation. This potential association has also been seen with the oral suspension and intravenous formulations in other studies [13, 14]. Albumin ≤3 g/dL was found to be associated with higher rates of subtherapeutic POSA concentrations at targets of ≥1.25 µg/mL and ≥1.8 µg/mL for oncology patients receiving a 300-mg oral DR-POSA formulation. Of the 47 patients with albumin ≤3 g/dL, only 19 (40.4%) reached POSA concentrations of ≥1.25 µg/mL, which was substantially less than the 75% reported in prior studies [5]. For a target of ≥1.8 µg/mL, which may be a target for more resistant pathogens, only 10 (21.3%) patients with albumin ≤3 g/dL met this target with 300-mg daily dosing [4]. While gender appeared to be associated with subtherapeutic levels in univariable analysis and has been studied for different target concentrations with the 300-mg tablet dosing, this was not demonstrated in multivariable analysis in this study [15, 16]. Other studies have shown diarrhea to be associated with subtherapeutic levels in multivariable analysis with 300-mg tablet dosing, but this was not seen in this study [16, 17]. Proton pump inhibitor use, diarrhea, and BMI ≥30 kg/m^2^ have been shown to be associated with lower POSA levels at other target concentrations, but this was not found for targets evaluated in this study [9, 18]. While this study was unable to demonstrate this finding, for POSA targets of ≥0.7 µg/mL, hypoalbuminemia has been associated with subtherapeutic levels in other studies [17]. This study found a similar finding at different POSA target concentrations that may be more appropriate for patients receiving therapy for IFI and a specific albumin level that may warrant consideration of higher upfront dosing. Posaconazole is >98% protein-bound, primarily to albumin, which may potentially explain this finding; however, patient factors are far more complex, and pharmacodynamic effects may exceed the predicted non-protein-bound drug concentration [19].

While this study was not designed to evaluate differences in mortality, given the high risk of mortality of IFI in oncology patients as well as higher risk of breakthrough IFI or treatment failures with subtherapeutic drug concentrations, there is a need to evaluate potential risk factors. Higher doses of POSA such as 400 mg/d have been shown to achieve higher median concentrations in hematologic malignancy patients and are generally well tolerated [20]. For this study, 15 patients receiving POS for treatment of IFI with concentrations <1.25 µg/mL had a subsequent increase in POSA dose. Of those, 12 had subsequent level evaluations, and 6 (50%) were found to be ≥1.25 µg/mL (median [IQR], 1.28 [0.70–1.63] µg/mL). In this study, POSA was stopped in 57 patients; however, in 29 this was due to death or transition to hospice care, with 14 potentially attributed to their IFI. Only 3 patients stopped therapy due to side effects attributed to POSA. This included 2 patients who developed transaminitis and 1 patient with potential adrenal insufficiency. Given the substantial delay from drug initiation until a concentration is known due to a need to achieve steady state and owing to the fact that many institutions do not have readily available in-house testing, there may be a delay in optimal POSA dosing in oncology patients receiving treatment for IFI.

The primary limitation of this study is its retrospective nature, which may increase variability between groups or lead to additional confounders that were not apparent. Generalizability may be limited, though 92 (93.9%) patients had hematologic malignancy. Of the 6 others, 2 had aplastic anemia and 1 hemophagocytic lymphohistiocytosis. Tissue concentrations may differ from plasma or serum POSA concentrations based on multiple clinical factors, but such sampling is not readily available or practical for routine patient care. While there is concern for increased mortality in patients with IFI with subtherapeutic levels of DR-POSA, controlled, prospective studies are needed to evaluate and characterize this risk.

Based on this study, in oncology patients with albumin ≤3 g/dL a higher initial dosing strategy for DR-POSA could be considered, particularly for patients with suspected IFI, with subsequent therapeutic drug monitoring and adjustment as appropriate. IBW ≥60 kg may be another population that requires consideration. Further prospective and controlled studies are required to confirm this finding, monitor for potential increased rates of adverse events, and evaluate the clinical efficacy of a higher initial dosing strategy.

## Supplementary Material

ofae185_Supplementary_Data

## Supplementary Data

Supplementary materials are available at *Open Forum Infectious Diseases* online. Consisting of data provided by the authors to benefit the reader, the posted materials are not copyedited and are the sole responsibility of the authors, so questions or comments should be addressed to the corresponding author.
