# Supplementary material for: Real-world Experience of Posaconazole Therapeutic Drug Monitoring in Oncology Patients: Clinical Implications of Hypoalbuminemia as a Predictor of Subtherapeutic Posaconazole Levels
Source: Open Forum Infect Dis. 2024 Mar 29;11(5):ofae185. doi: 10.1093/ofid/ofae185 (PMC11055390; doi:10.1093/ofid/ofae185)
Supplement: ofae185_Supplementary_Data [file ofae185_supplementary_data.docx]

110 patients with posaconazole level obtained at Moffitt Cancer Center from 06/01/2021 – 07/31/2023

3 patients excluded for level obtained < 4 hours from administration

3 patients receiving intravenous formulation posaconazole

2 patients not taking medication when level drawn

1 patient receiving posaconazole solution formulation

1 patient receiving 400mg dosing

1 patient excluded for level drawn on day of drug initiation

1 patient receiving 100mg three times daily dosing

98 patients included in the analysis

Supplemental Figure 1: Identification of study cohort of patients receiving delayed-release posaconazole at 300mg orally per day dosing from 06/2023-07/2023 with therapeutic drug monitoring

Supplemental Table 1: Risk factors for subtherapeutic posaconazole concentrations for a target concentration ≥ 0.7 µg/mL

| **Risk Factor** | **Posaconazole Level < 0.7 µg/mL (n=19)** | | **Posaconazole Level ≥ 0.7 µg/mL (n=79)** | | ***P*-value** | **Multivariable logistic regression** | |
| --- | --- | --- | --- | --- | --- | --- | --- |
|  |  |  |  |  |  | **OR (95% CI)** | ***P*-value** |
| Age, years (IQR) | 61.00 (57.00-70.00) |  | 63.00 (53.00-68.00) |  | 0.780 |  |  |
| Weight, kg (IQR) | 89.90 (67.90-109.00) |  | 80.20 (66.70-89.90) |  | 0.070 |  |  |
| Weight >90kg | 9 | 47.4% | 19 | 24.1% | **0.043** | 0.45 (0.15-1.35) | 0.154 |
| Height, cm (IQR) | 177.50 (168.00-178.00) |  | 172.00 (164.00-180.00) |  | 0.338 |  |  |
| IBW, kg (IQR) | 72.28 (59.63-73.18) |  | 67.30 (57.06-74.99) |  | 0.364 |  |  |
| IBW ≥ 60kg | 14 | 73.7% | 56 | 70.9% | 0.808 |  |  |
| BMI, kg/m^2^ (IQR) | 30.24 (23.49-34.79) |  | 26.28 (23.71-29.55) |  | 0.265 |  |  |
| Female Gender | 6 | 31.6% | 27 | 34.2% | 0.830 |  |  |
| AML | 8 | 42.1% | 39 | 49.4% | 0.569 |  |  |
| Myeloid Malignancy | 14 | 73.7% | 54 | 68.4% | 0.651 |  |  |
| AlloSCT | 14 | 73.7% | 35 | 44.3% | **0.021** | 0.37 (0.12-1.19) | 0.096 |
| GI aGVHD | 1 | 5.3% | 9 | 11.4% | 0.681 |  |  |
| Interacting drug - any | 15 | 78.9% | 67 | 84.8% | 0.506 |  |  |
| PPI | 12 | 63.2% | 39 | 49.4% | 0.280 |  |  |
| Antacid, single agent | 15 | 78.9% | 65 | 82.3% | 0.746 |  |  |
| Diarrhea | 8 | 42.1% | 13 | 16.5% | **0.026** | 0.34 (0.11-1.06) | 0.064 |
| Albumin ≤ 3 g/dL | 9 | 47.4% | 38 | 48.1% | 0.954 |  |  |

Abbreviations: IQR, interquartile range; IBW, ideal body weight; BMI, body mass index; AML, acute myeloid leukemia; AlloSCT, allogeneic stem cell transplant; GI aGVHD, acute gastrointestinal graft-versus-host disease; PPI, proton pump inhibitor
